# Destination image, nostalgic feeling, flow experience and agritourism: An empirical study of Yunling Tea Estate in Anxi, China

**DOI:** 10.3389/fpsyg.2022.954299

**Published:** 2022-09-08

**Authors:** Sunbowen Zhang, Jingxuan Liang, Yongqiang Ma, Youcheng Chen, Qiaohua He

**Affiliations:** ^1^Anxi College of Tea Science, Fujian Agriculture and Forestry University, Quanzhou, China; ^2^College of Business & Management, Xiamen Huaxia University, Xiamen, China

**Keywords:** destination image, nostalgic feeling, flow experience, tea estate tourism, cultural memory

## Abstract

This study introduces destination image, nostalgic feeling, and flow experience into tea estate tourism and constructs a theoretical model that includes destination image, nostalgic feeling, flow experience, cultural identity, and tourists’ behavioral intention. Then, an empirical study is conducted with tourists at Yunling Tea Estate in Anxi, China. The results show that all hypotheses are supported except the hypothesis pertaining to the significance of the influence of flow experience on behavioral intention, which is not supported. The model includes eight mediating effects and one moderating effect that is influenced by cultural memory.

## Introduction

Since its introduction into the field of tourism in the 1970s, the concept of image has become a research hotspot. The image of a tourism destination is usually considered a system of interactions among tourists’ perceptions, opinions, emotions, and expectations of a destination after a trip (Tasci et al., 2007), similar to the concept of mental representation in cognitive psychology, and is a subjective interpretation of reality by tourists (Seyhmus et al., 1999; Bigne et al., 2001). In the current field of tourism research, image management has become one of the most important responsibilities of destination marketers as destination image has an extremely important impact on tourists’ travel decisions and destination choices (Chi, 2010; Nunkoo et al., 2013). As a general focus in the academic community, many studies on destination image have focused on image formation (Martin and Bosque, 2008). Destination image theory has also been used to study different tourism experiences and behaviors such as the images of heritage sites and theme parks (Zhao et al., 2013; Szubert et al., 2021; Zhu et al., 2022).

Continuous advancements in China’s rural revitalization strategy have facilitated the integration of primary, secondary and tertiary industries in China’s rural areas, resulting in a continuous positive impact on the agritourism industry. The image of agritourism destinations represented by tea estates has been receiving increasingly more attention from tourists (Lei and Zhou, 2020). As a brand-new concept, tea estates not only have the cultural connotation of agricultural cultural heritage but have also become a new type of social-media-worthy tourism subject, providing characteristic tea culture products and services to tourists.

When tourists acquire a good destination image of a tea estate, are their travel experience and behavioral intention enhanced? Through what mechanism does the image of a tea estate influence behavioral intention? A literature review of previous studies provides no answers to the above questions. Therefore, the possible contributions of this study are as follows. First, previous studies involving destination image have mainly focused on heritage and cultural tourism destinations (Su et al., 2020a, b); this study takes tea estates as the research object to enrich tourism destination image research. Second, psychological perception plays an important role in tourism experience (Zhang et al., 2022). However, there is very limited research involving the psychological perception dimension of tourists in studies on tea estates and agricultural estates. Therefore, this study also helps to enrich the research on the psychological perception of tourists. Third, this is the first study on the tourism destination image of tea estates, and the findings may provide a better understanding of the attitude and behavioral intention of tea estate tourists as well as provide a feasible marketing approach for tea estate managers.

## Literature review and hypothesis

### Tea estates

The earliest agricultural estates were created with the feudal system (Gizicki-Neundlinger et al., 2017). With development across time, agricultural estates gradually transformed into a crossover industry combining agriculture and tourism by utilizing agricultural resources and agricultural landscapes. As a new practice among agricultural estates in recent years, there is no unified definition of tea estates. Tea estates are projects built using a business model that combines French wine estates and Chinese customs. Scholars’ definition of tea estates is to emphasize that tea estates can meet the richness of tourists’ perceived experience, and provide tourists with wine and food tasting, manor environment enjoyment, cultural exchange, lifestyle integration and other elements (Sparks, 2007). Therefore, this study considers tea estates as multifunctional agricultural estates that combine tourism and the tea industry by relying on the advantages of tea landscapes and agricultural cultural heritage and integrating the planting, production, marketing, culture, tourism, and scientific research of tea.

### Destination image

Since the concept of tourism destination image was introduced in the 1970s, tourism destination image research has been a hot topic in and focus of tourism academia. Currently, there are a variety of viewpoints, such as the “attribute theory,” “summation theory,” “holistic theory,” and “system theory,” regarding the definition of tourism destination image in academia. The root cause of the different viewpoints lies in the inconsistent understanding of the internal structure and organization of tourism destination image. This study adopts the “system theory” viewpoint, i.e., the image of a tourism destination is a system of interactions among tourists’ perceptions, opinions, emotions, and expectations of a destination after travel (Tasci et al., 2007), similar to mental representation in cognitive psychology, and is a subjective interpretation of reality by tourists (Seyhmus et al., 1999; Bigne et al., 2001).

From a theoretical perspective, destination image research has always been a hot topic in tourism research, with the research focusing on measuring tourism image (Stepchenkova and Zhan, 2013), tourism image marketing (Kock et al., 2016), factors that influence tourism image perception (Molinillo et al., 2018), and tourism image perception behavior models (Stylidis et al., 2017). However, many studies have paid too much attention to changes in destination image and have neglected to explore the cognitive processes by which tourists form and change elements of tourism destination image. From a practical perspective, previous studies have shown that the image of a tourism destination can influence tourist behavior before, during, and after a trip (Agapito et al., 2013), positively or negatively affecting tourists’ satisfaction with the destination and their intention to revisit (Chen and Chen, 2010). However, most destination studies have different starting points, and thus, destination image results are hardly representative and generalizable (Su et al., 2020a, b).

Balakrishnan et al. (2021) discuss the relationship between destination image and tourists’ flow experience. Chang et al. (Chang and Chiang, 2022) find that destination image has a significant positive impact on tourists’ psychological distance. Other studies further support these findings, suggesting that destination image can positively influence consumers’ flow experience (Kim and Hall, 2019; Chang, 2021).

Akgun et al. and Kan et al. investigate the relationship between destination image and tourists’ nostalgia (Akgun et al., 2020; Kan et al., 2021). Li (Li et al., 2021) argues that destination image, such as authentic food and atmosphere, can trigger nostalgia. Shi (Shi et al., 2021) experimentally verifies that a destination image constructed by both genuine and artificial approaches can trigger nostalgia but that a genuine destination image has a stronger and more positive impact than does an artificial image.

Numerous studies have revealed that destination image can have a positive impact on tourist identity. For example, Lee (Lee et al., 2021) notes the correlation between destination image and cultural identity and finds that the image of indigenous people at a tourism destination can positively influence the cultural identity of tourists. Tsaur (Tsaur et al., 2016) further supports the findings of previous research by arguing that destination image, quality, and culture can promote tourists’ sense of identity.

Therefore, this study argues that because Yunling Tea Estate is both a social-media-worthy destination and has the cultural characteristics of a state-run old tea estate and agricultural cultural heritage site, tourist involvement in activities is high, thus prompting a series of psychological perceptions. On this basis, the following hypotheses are proposed:

*H1*: Destination image can positively influence tourists’ flow experience.

*H2*: Destination image can positively influence tourists’ nostalgic feeling.

*H3*: Destination image can positively influence tourists’ cultural identity.

### Flow experience

Flow is a psychological state, an optimal inner state in which an individual feels engaged and experiences a high degree of pleasure (Decloe et al., 2009). When environmental conditions meet tourists’ personal goals, tourists engage in activities, take control of situations, and enter a state of flow (Wu and Liang, 2011; Wöran and Arnberger, 2012).

A previous study has confirmed the relationship between flow experience and cultural identity and verified the positive impact of flow experience on tourists’ sense of identity (Perez-Vega et al., 2018). As Bonaiuto (Bonaiuto et al., 2016) maintains, flow experience at a favorite destination is widely reported to be caused by a series of self-defining activities and can positively and significantly influence correlation identity, regardless of gender or age.

Many studies have confirmed the relationship between flow experience and behavioral intention and revealed that flow experience can have a positive impact on consumer behavioral intention. For example, Hsu (Hsu et al., 2012), using the data collected from 395 customers of online shopping stores, verifies that flow experience is significantly positively correlated with online shopping behavior. From the perspective of electronic product purchases, Zhou (Zhou, 2012) discovers that flow experience and trust both determine the intention to use, further supporting the previous research findings. Therefore, the following hypotheses are proposed:

*H4*: Flow experience can positively influence tourists’ cultural identity.

*H5*: Flow experience can positively influence tourists’ behavioral intention.

### Nostalgic feeling

“Nostalgia” is used to describe the emotional reminiscence about an entity or environment (Chen et al., 2014). Nostalgia has long been associated with feelings such as homesickness (Hofer, 1934). However, across time, nostalgia has also become linked with psychological and psychiatric disorders and is understood as a highly selective view of the past and as positive feelings about a particular past (Fine and Davis, 1980), leading to an increase in research on the underlying psychology (Wildschut et al., 2018).

Many studies have confirmed the relationship between nostalgia and cultural identity and demonstrated that nostalgia can have a positive impact on tourists’ cultural identity (Brown, 2010). For example, Zou (Zou et al., 2021) uses a hybrid approach to examine the changes in the emotions of Chinese diaspora tourists towards their ancestral hometowns, arguing that nostalgic memories positively influence local identity and social ties.

Some scholars have explored the relationship between nostalgia and behavioral intention and demonstrated that nostalgia positively influences tourists’ behavioral intention (Kessous et al., 2015). For example, Fan (Fan et al., 2020) finds that nostalgia can enhance consumers’ social ties, thereby prompting the maintenance of a sense of connection with the group and increasing their preference for majority-approved choices in their subsequent product choices. Therefore, the following hypotheses are proposed:

*H6*: Nostalgic feeling can positively influence tourists’ cultural identity.

*H7*: Nostalgic feeling can positively influence tourists’ behavioral intention.

### Cultural identity

Tourism has been shown to promote the development of self-identity (Palmer, 2005). Cultural identity “the individual tourist’s self-constructed understanding of his or her cultural membership” (Phinney, 2005). Cultural identity enables individual tourists to connect with others and define self-concepts. The stronger is an individual’s cultural identity, the more loyal he or she is to the values and norms of the group (Alden et al., 2010). Numerous studies have confirmed the relationship between cultural identity and behavioral intention. For example, He (He and Wang, 2015) explores the impact of Chinese cultural identity and consumer ethnocentrism on the preference for and purchase of domestic vs. imported brands and finds that cultural identity enhances the preference for and purchase of domestic brands. The conclusions by Zhang (Zhang et al., 2020) further support those of previous studies, i.e., in tourism development, tourists and local residents have reached a common cognitive basis for the Kunqu Opera through cultural identity, thereby leading consumer behavior to drive the continuous development of the Kunqu Opera. Therefore, based on review of previous literature, the following hypothesis is proposed:

*H8*: Cultural identity can positively influence tourists’ behavioral intention.

### Behavioral intention

Behavioral intention refers to the subjective probability that people exhibit a certain behavior (Oliver, 1997). Early research in the field of consumer behavior considers behavioral intention as an abstract concept that includes four categories: intention to buy, intention to pay a premium, intention to repurchase, and intention to recommend (Zeithaml et al., 1996). Subsequently, the discussion of tourists’ behavioral intention in the tourism field has mainly focused on the intention to revisit (Wu and Li, 2017) and the intention to recommend (Sharma and Nayak, 2018). As Davis (Davis et al., 1989) points out, tourists’ behavioral intention refers to the specific behaviors or behavioral tendencies, such as the intention to travel, intention to recommend, and intention to revisit, that tourists may adopt after learning about a tourism destination. This understanding of behavioral intention has also been verified by many studies (Kim and Kim, 2018; Lai et al., 2018).

### Mediating effect

As found in the above studies, destination image (Loureiro and Kaufmann, 2011; Jiang et al., 2017), flow experience (Bonaiuto et al., 2016; Perez-Vega et al., 2018), and nostalgia (Brown, 2010; Zou et al., 2021) are antecedent variables of cultural identity, and behavioral intention is the outcome variable of cultural identity (He and Wang, 2015; Yen et al., 2017; Zhang et al., 2020). Therefore, we have reason to believe that cultural identity may play a mediating role in the relationship between destination image, flow experience, and nostalgic feeling with behavioral intention.

As found in the above studies, destination image is an antecedent variable of flow experience and nostalgic feeling, and behavioral intention is an outcome variable of flow experience and nostalgia (Li et al., 2021; Shi et al., 2021). Therefore, it is reasonable to believe that flow experience and nostalgic feeling may play a mediating role in the relationship between destination image and behavioral intention. Furthermore, destination image can influence behavioral intention by influencing the chain mediating role of flow experience and cultural identity and of nostalgia and cultural identity.

As found in the above studies, destination image is an antecedent variable of flow experience and nostalgia, and cultural identity is an outcome variable of flow experience (Bonaiuto et al., 2016; Perez-Vega et al., 2018) and nostalgia (Brown, 2010; Zou et al., 2021). Therefore, we have reason to believe that flow experience and nostalgia may play a mediating role in the relationship between destination image and cultural identity. Therefore, the following hypotheses are proposed:

*H9a*: Cultural identity plays a mediating role in the relationship between destination image and behavioral intention.

*H9b*: Cultural identity plays a mediating role in the relationship between flow experience and behavioral intention.

*H9c*: Cultural identity plays a mediating role in the relationship between nostalgic feeling and behavioral intention.

*H10a*: Flow experience plays a mediating role in the relationship between destination image and behavioral intention.

*H10b*: Flow experience plays a mediating role in the relationship between destination image and cultural identity.

*H10c*: Flow experience and cultural identity play a chain mediating role in the relationship between destination image and behavioral intention.

*H11a*: Nostalgic feeling plays a mediating role in the relationship between destination image and behavioral intention.

*H11b*: Nostalgic feeling plays a mediating role in the relationship between destination image and cultural identity.

*H11c*: Nostalgic feeling and cultural identity play a chain mediating role in the relationship between destination image and behavioral intention.

### Moderating effect

**Figure 1:**
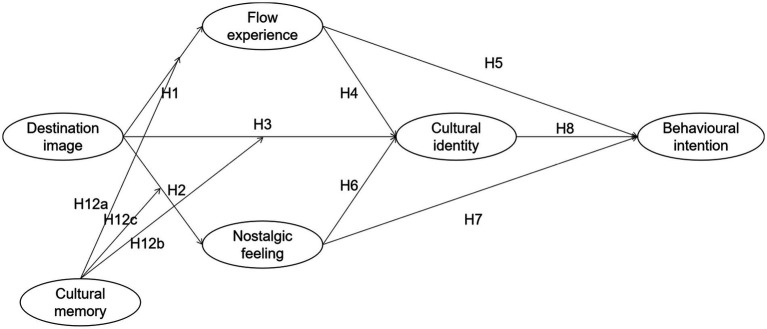
The theoretical model of this study.

Cultural memory is the spatial aggregation of past cultures through images, texts, cultural relics, and historical sites, which are then reconstructed in the collective consciousness of the public (Meusburger et al., 2011). Cultural tourism experiences offer the possibility for the creation of personal memory and even facilitate the formation of cultural memory (Withers, 2005). The above evidence shows that destination image can have a positive impact on flow experience, cultural identity, and nostalgic feeling. However, in addition to exploring the direct and mediating effects of destination image on flow experience, cultural identity, and nostalgic feeling, the indirect effects of destination image on flow experience, cultural identity, and nostalgic feeling are also worth studying. By analyzing the results of a sample of 651 tourists, Li et al. (Li and Liu, 2020) find that culture enhances tourists’ destination image through creative experience and cultural memory. From the perspective of film image, Kim (Kim, 2012) suggests that faded memory may cause film destinations to lose their appeal. Therefore, we believe that cultural memory may have a moderating effect on the relationship of tea estate image with flow experience, cultural identity, and nostalgic feeling (Figure 1). Therefore, the following hypotheses are proposed:

*H12a*: Cultural memory plays a moderating role between destination image and flow experience.

*H12b*: Cultural memory plays a moderating role between destination image and nostalgic feeling.

*H12c*: Cultural memory plays a moderating role between destination image and cultural identity.

## Research methods

### Introduction to the study area

**Figure 2:**
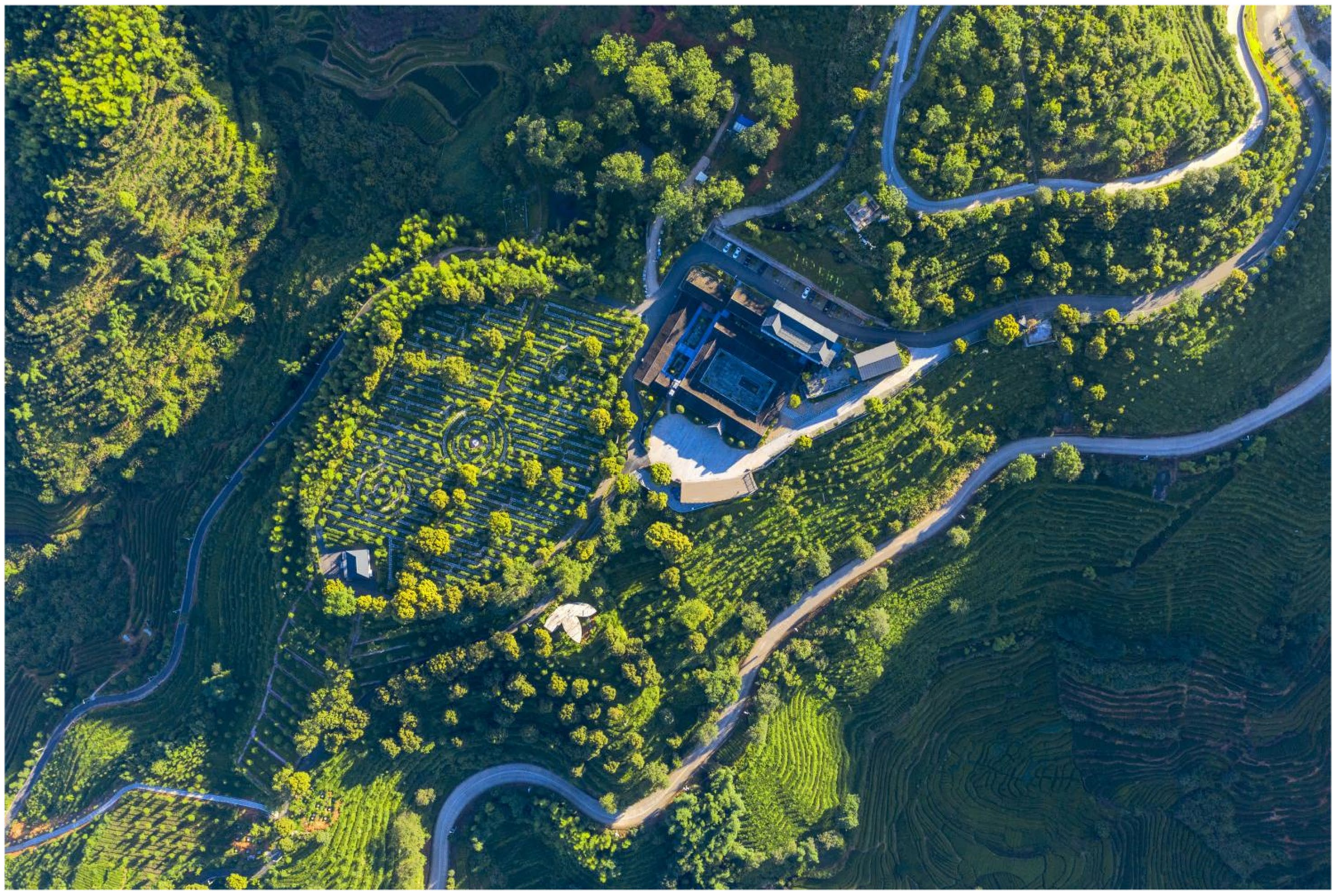
Anxi Yunling Tea Estate. Adapted/Reproduced with permission from Anxi Yunling Tea Estate, available at: https://commons.wikimedia.org/wiki/File:Anxi_Yunling_Tea_Estate.jpg.

**Figure 3:**
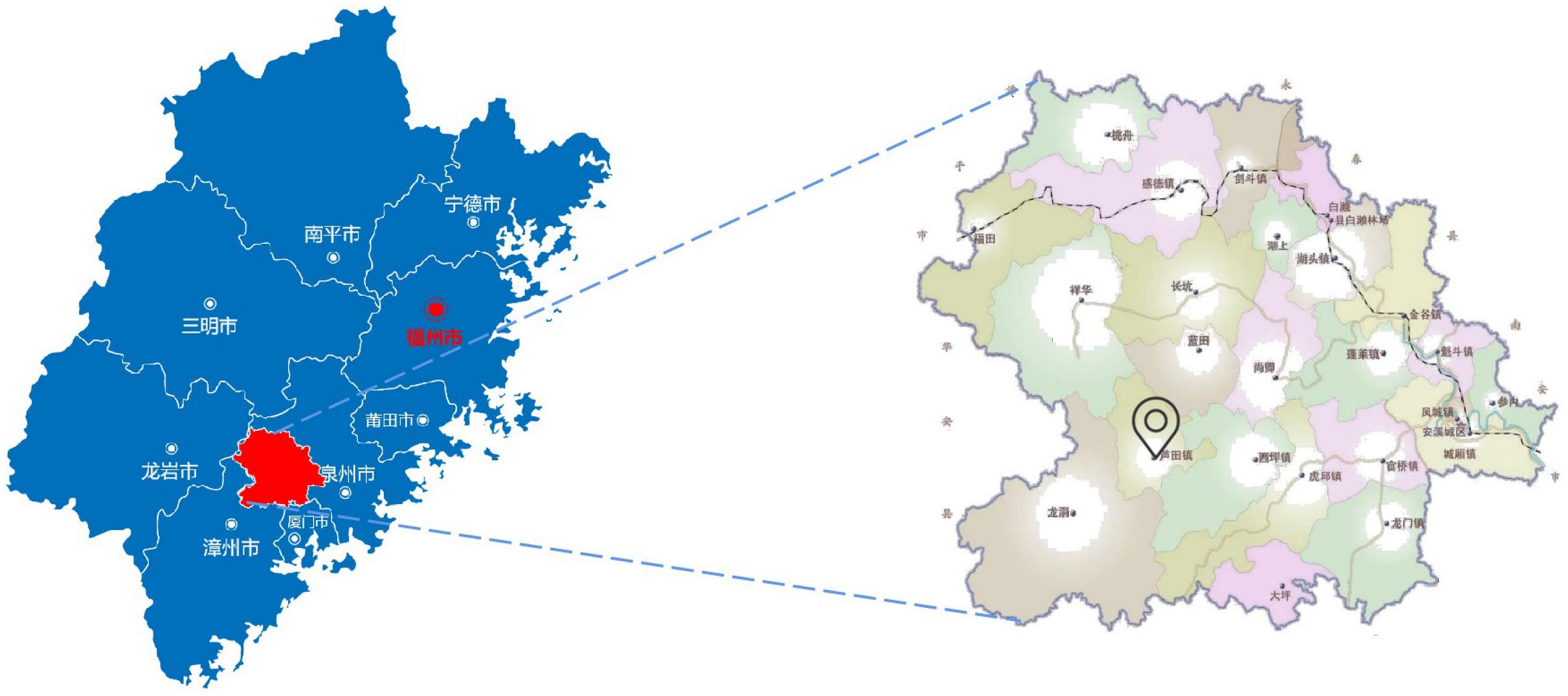
Anxi Yunling Tea Estate location conditions.

Yunling Tea Estate is located in Anxi County, China (117°53′28.95″E, 25°1′59.47″N), and the core area of a globally important agricultural heritage system (GIAHS). Currently, it has an existing land area of nearly 270.78 hm^2^, which includes a tea plantation area of about 92.7 hm^2^ and a forest land area of 160.08 hm^2^. In recent years, Yunling Tea Estate has fully exploited the local characteristic resources and used “materials in memory” to transform the surrounding landscapes (such as ancient houses in southern Fujian and old courtyard houses) in a natural and simple style to inspire a strong tea culture, to help impart feeling of nostalgia, and to build a tea industry complex that integrates tea production and processing, tea culture dissemination, research and development innovation, tourism and sightseeing, and leisure experiences. Currently, Yunling Tea Estate has become a demonstration site for the integration of three industries in China’s modern agricultural industrial parks, more importantly, a representative agricultural development project in Fujian and in China, with a brand effect (Figures 2, 3).

### Questionnaire design

The questionnaire for this study consists of two parts. The first part includes 27 variables from the six constructs in the research model. All variables are measured using a seven-point Likert scale, ranging from 1 (strongly disagree) to 7 (strongly agree). The second part is designed to collect background information from the respondents (Supplementary Table A1 in Appendix A). The destination image scale is derived from Jalilvand et al. (2012), the psychological distance scale is derived from Choi et al. (Choi et al., 2007), the nostalgia scale is derived from Davari et al. (2017), the cultural identity scale is derived from Tonge et al. (2015), the behavioral intention scale is derived from Su et al., 2020a, b, and the cultural memory scale is derived from Lai et al. (2021). All scales were modified to fit the actual situation at the tea estate and were professionally translated into Chinese and then back into English to verify the consistency, ensure the reliability of the questionnaire.

### Data collection

From January 1, 2021, to January 27, 2021, a pre-study was conducted with 50 tourists at Yunling Tea Estate in Anxi to determine the validity of the questionnaire content. All respondents indicated that they were able to fully understand all the items in the questionnaire; therefore, there was no need for further questionnaire modification.

The formal questionnaire for this study was distributed offline using a convenience sampling method. The data were collected from March 16, 2021, to July 20, 2021. Considering factors such as an increase in the number of tourists to the tea estate and wider coverage during weekends and holidays to increase the representativeness of the sample data, this study focused on weekends and holidays (such as Labour Day and Dragon Boat Festival) in the 4 months of the study period. On each of these days, the research team chartered a bus to Yunling Tea Estate in Anxi and invited tourists to participate in the questionnaire survey from 10:00 to 16:00. The team members first introduced the study to the respondents and then, upon obtaining their consent, distributed the questionnaire. It took the tourists an average of 15 min to complete the questionnaire. A total of 309 questionnaires were distributed and, after excluding 32 invalid questionnaires, 277 valid questionnaires were collected, for a recovery rate of 89.64%.

## Survey results

In this study, PLS-SEM is used for the following reasons. First, compared with covariance-based structural equation modelling (CB-SEM), partial least squares SEM (PLS-SEM) does not have a high requirement with regard to the assumption of a normal distribution of data (Hair et al., 2019). The study area has social-media-worthy characteristics, which are more in line with the preferences of young people; therefore, the study subjects include a high concentration of young people. Second, PLS-SEM can be used for data analysis of small samples (Hair et al., 2016). The model in this study contains six concepts with many indicators and complex relationships, and the sample size for this study is not large enough. In addition, the purpose of this study is to investigate the influence of tea estate image on tourists’ flow experience, nostalgic feeling, cultural identity, and behavioral intention, not a simple comparison of theories and models (Hair et al., 2017). In summary, PLS-SEM is the most suitable analytical method for this study. Therefore, SmartPLS 3.3.3 is used for data analysis (Schlittgen et al., 2015).

### Measurement model evaluation

Common method bias test. This test is used to assess the presence of common method bias in the obtained data, an error that can directly affect the accuracy of the recovered data. A single-factor test is used to measure common method bias in the data. The results indicate that the explained variance in a single factor is less than 50% of the total explained variance; therefore, common method variation in the data is within the acceptable range (Harman, 1976).

Reliability and validity tests. In accordance with the research procedure recommended by Hair (Hair et al., 2019), this study separately verifies the reliability and validity of the measurement model. As seen in Table 1, the factor loading of each of the 27 variables is above 0.7. For each variable, the Cronbach’s *α* and the combined reliability (CR) are both higher than 0.7, and the average variance extracted (AVE) is above 0.5, indicating that the measurement model has good reliability and convergent validity (Hair et al., 2017). In addition, as seen in Table 2, this study further tests discriminant validity based on the Fornell–Larcker criterion, and the data obtained are consistent with acceptable discriminant validity (Henseler et al., 2015; Hair et al., 2017).

**Table 1:** Results of the confirmatory factor analysis (*N* = 277).

Measurement item	Mean	SD	Factor loading	VIF	Cronbach’s alpha	CR	*R* ^2^	*Q* ^2^	AVE
**Destination image**									
DI1 Yunling Tea Estate is an area with a great cultural atmosphere.	5.444	1.112	0.845	2.356	0.873	0.908	/	/	0.664
DI2 The art at Yunling Tea Estate is fascinating.	5.401	1.076	0.809	2.115					
DI3 Yunling Tea Estate has beautiful natural scenery.	5.776	1.058	0.824	2.516					
DI4 Yunling Tea Estate is a place for self-reflection.	5.812	1.075	0.831	2.540					
DI5 The infrastructure of Yunling Tea Estate is very good.	5.162	1.127	0.763	1.604					
**Flow experience**									
FE1 Participating in the tea estate tour was very interesting.	5.162	1.057	0.842	1.844	0.862	0.916	0.574	0.436	0.784
FE2 I was completely relaxed when I participated in the tea estate tour.	5.292	1.077	0.917	2.774					
FE3 In general, I am very happy with the tea estate tour.	5.458	1.042	0.896	2.506					
**Nostalgic feeling**									
NF1 The Yunling Tea Estate tour allowed me to learn about its past.	4.159	1.545	0.701	2.058	0.849	0.887	0.427	0.221	0.568
NF2 The Yunling Tea Estate tour allowed me to recognize the wisdom and strength of the workers in tea villages.	4.35	1.331	0.713	2.088					
NF3 The Yunling Tea Estate tour made me feel sorry about the experiences at the old tea factory.	4.513	1.323	0.785	2.039					
NF4 The Yunling Tea Estate tour helped me recall the long history of tea culture in Anxi.	4.982	1.182	0.790	2.174					
NF5 The Yunling Tea Estate tour helped me strengthen my belief in preserving agricultural cultural heritage.	4.939	1.174	0.803	2.252					
NF6 The Yunling Tea Estate tour made me feel nostalgic.	5.148	1.080	0.723	1.520					
**Cultural identity**									
CI1 Yunling Tea Estate is part of my life.	4.957	1.094	0.807	2.473	0.900	0.923	0.772	0.504	0.668
CI2 I like Yunling Tea Estate very much.	4.812	1.159	0.839	2.676					
CI3 I strongly identify with Yunling Tea Estate.	5.022	1.111	0.865	2.840					
CI4 Yunling Tea Estate is very special to me.	5.043	1.210	0.844	2.737					
CI5 The Yunling Tea Estate tour taught me a lot about tea culture.	5.274	1.093	0.782	2.486					
CI6 Yunling Tea Estate has a great impact on me.	5.292	1.074	0.761	2.409					
**Behavioral intention**									
BI1 I would recommend Yunling Tea Estate to my friends or relatives.	5.101	1.116	0.896	3.030	0.891	0.925	0.709	0.526	0.755
BI2 I would be very happy to visit Yunling Tea Estate again if I have the opportunity.	5.152	1.074	0.886	2.920					
BI3 I would be willing to pay a higher price for the Yunling Tea Estate tour.	4.368	1.349	0.806	1.888					
BI4 I always speak highly of Yunling Tea Estate to others.	4.794	1.182	0.885	2.580					
**Cultural memory**									
CM1 I have fond memories of Yunling Tea Estate.	5.39	1.030	0.911	3.063	0.889	0.931	/	/	0.819
CM2 I will never forget my experience at Yunling Tea Estate.	5.274	1.046	0.877	2.113					
CM3 This tour experience has left me with a variety of memories and benefits.	5.35	1.042	0.926	3.445					

**Table 2:** Discriminant validity test.

Measurement item	Destination image	Flow experience	Nostalgic feeling	Cultural identity	Behavioral intention	Cultural memory
Destination image	**0.815**					
Flow experience	0.529	**0.885**				
Nostalgic feeling	0.487	0.663	**0.754**			
Cultural identity	0.583	0.760	0.818	**0.817**		
Behavioral intention	0.561	0.678	0.796	0.803	**0.869**	
Cultural memory	0.467	0.723	0.581	0.640	0.568	**0.905**

AVE, average variance extracted; bold font, square-root of the AVE.

Collinearity test. Regarding the presence of multicollinearity, the variance inflation factor (VIF) of exogenous variables (all in the range of 1.520–3.445) is less than the recommended maximum value of 5.0. Therefore, there is no serious collinearity problem in this study (O’Brien, 2007).

### Structural equation model

**Figure 4:**
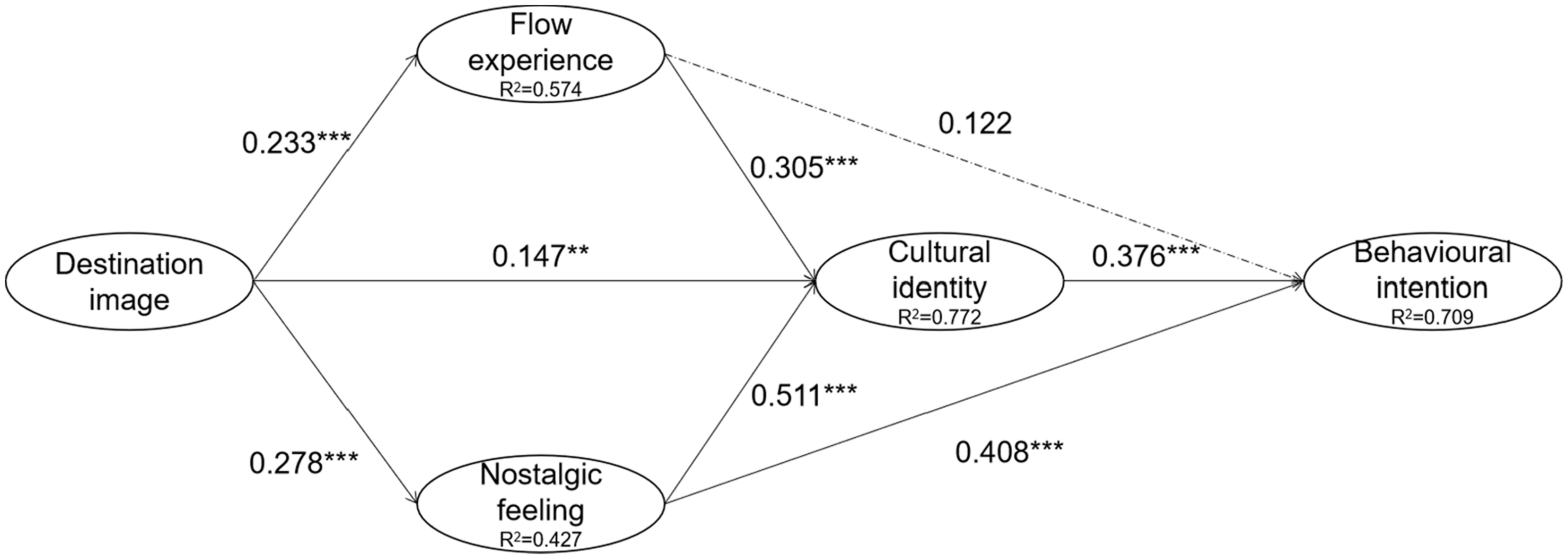
Structural model and path coefficients. ^***^p < 0.001, ^**^p < 0.01, ^*^p < 0.05, solid line means significant, and dashed line means not significant.

The statistical significance of the model is tested with 5,000 samples using the bootstrapping method (Hair et al., 2016). As seen in Table 3 and Figure 4, the coefficients of determination (*R*^2^) of the endogenous constructs are all range from 0.4 to 0.8, indicating that the structural equation model has a moderate influence capability (Hair et al., 2016). *Q*^2^ is used to measure the correlation of the influence of endogenous constructs. In this study, all constructs have a *Q*^2^ greater than 0.1, indicating the influence correlation of the model (Evermann and Tate, 2016). As seen from the PLS-SEM analysis results in Table 3, destination image can positively and significantly influence flow experience (*β* = 0.233, *t* = 4.152, *p* = 0.000), nostalgic feeling (*β* = 0.278, *t* = 5.50, *p* = 0.000), and cultural identity (*β* = 0.147, *t* = 3.449, *p* = 0.001); flow experience can positively and significantly influence cultural identity (*β* = 0.305, *t* = 5.816, *p* = 0.000); nostalgic feeling can positively and significantly influence cultural identity (*β* = 0.511, *t* = 10.426, *p* = 0.000) and behavioral intention (*β* = 0.408, *t* = 6.786, *p* = 0.000); and cultural identity can positively and significantly influence behavioral intention (*β* = 0.376, *t* = 5.449, *p* = 0.000). Therefore, hypotheses H1, H2, H3, H4, H6, H7, and H8 are supported. In addition, the SmartPLS analysis results suggest that the relationship between flow experience and tourists’ behavioral intention (H5) is not significant (*β* = 0.122, *t* = 1.656, *p* = 0.098).

**Table 3:** Path coefficients.

Path	Path coefficient	*t*-value	*f*-square	*p*-value	Supported
H1:DI → FE	0.233	4.152	0.098	0.000	Yes
H2:DI → NF	0.278	5.507	0.106	0.000	Yes
H3:DI → CI	0.147	3.449	0.064	0.001	Yes
H4:FE → CI	0.305	5.816	0.150	0.000	Yes
H5:FE → BI	0.122	1.656	0.021	0.098	No
H6:NF → CI	0.511	10.426	0.563	0.000	Yes
H7:NF → BI	0.408	6.786	0.187	0.000	Yes
H8:CI → BI	0.376	5.449	0.119	0.000	Yes

*f* ^2^ is calculated to assess whether the omitted constructs have a substantial impact on the endogenous constructs. The results show that *f*  ^2^ for significant paths ranges from 0.021 to 0.563, indicating that the ranges of influence of tea estate image on tourists’ flow experience, nostalgic feeling, cultural identity, and behavioral intention change from small to large (Cohen, 1990; Zhu et al., 2022).

### Mediating effect test

As seen in Table 4, nostalgic feeling plays a partial mediating role between destination image and behavioral intention, cultural identity plays a partial mediating role between flow experience and behavioral intention, nostalgic feeling plays a partial mediating role between destination image and cultural identity, and cultural identity plays a partial mediating role between nostalgic feeling and behavioral intention. Therefore, hypotheses H9a, H9b, H9c, H10b, H10c, H11a, H11b, and H11c are supported, while H10a is not supported.

**Table 4:** Mediating effect test.

Path	Original sample	Standard error	*t*-value	*p*-value	Supported
H9a:DI → CI → BI	0.055	0.020	2.763	0.006	Yes
H9b:FE → CI → BI	0.115	0.026	4.418	0.000	Yes
H9c:NF → CI → BI	0.192	0.041	4.744	0.000	Yes
H10a:DI → FE → BI	0.028	0.022	1.318	0.188	No
H10b:DI → FE → CI	0.071	0.022	3.163	0.002	Yes
H10c:DI → FE → CI → BI	0.027	0.009	3.026	0.002	Yes
H11a:DI → NF → BI	0.114	0.027	4.164	0.000	Yes
H11b:DI → NF → CI	0.142	0.029	4.864	0.000	Yes
H11c:DI → NF → CI → BI	0.054	0.015	3.636	0.000	Yes

### Moderating effect test

**Figure 5:**
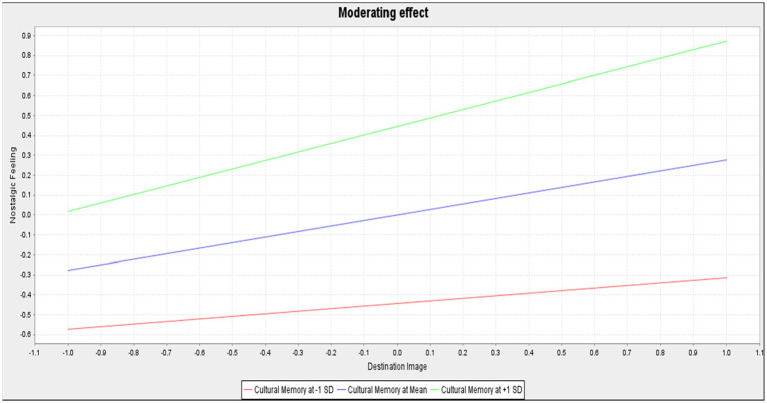
The moderating effect of cultural memory in destination image on nostalgia.

As seen in Table 5 and Figure 5, only hypothesis H12b is supported, indicating the magnitude of the moderating effect of cultural memory on nostalgic feeling (*β* = 0.149, *t* = 3.291, *p* = 0.001). The results indicate that cultural memory has a significant positive moderating effect on the positive correlation between destination image and nostalgic feeling. In other words, the more scenic areas with cultural memory that the tea estate has, the higher the feelings of nostalgia, and vice versa.

**Table 5:** Moderation effect test.

		Path coefficient	*t*-value	*f*-square	*p*-value	Supported
H12a	DI → FE (Moderating effect)	−0.056	0.724	0.011	0.469	No
H12b	DI → NF (Moderating effect)	0.149	3.291	0.052	0.001	Yes
H12c	DI → CI (Moderating effect)	0.012	0.357	0.001	0.721	No

## Conclusion and discussion

### Conclusion

The development of agritourism in the context of local conditions is an important measure to cultivate new industries and new business forms of agriculture in rural areas, which can promote the integration of rural industries and thus facilitate the realization of rural revitalization strategies. As an important carrier for the development of leisure agricultural experiences based on the tea industry in China’s important tea areas, tea estates are a special feature of the current development of leisure agriculture in China (Mao et al., 2019). The aim of this study was to discuss the relationship between the tourism destination image of a tea estate, consumer psychological perceptions (flow experience, cultural identity, and nostalgia), and post-trip behavioral intention. The results indicate that among all the direct effects, all hypotheses are supported except H5 (FE → BI). In addition, in terms of the mediating effect, all hypotheses hold except H10a (DI → FE → BI), and in terms of the moderating effect, only H12b (DI → NF) is supported; the other hypotheses are not.

### Discussion

Based on the destination image perspective, this study constructs a theoretical model for the influence of destination image of a tea estate on tourists’ attitude towards tourism to explore how destination image influences tourists’ attitude towards tea estate tourism through flow experience, nostalgic feeling, and cultural identity. A total of 16 out of 20 hypotheses are supported.

First, the relationships of the destination image of the tea estate with tourists’ flow experience (H1), nostalgic feeling (H2), and cultural identity (H3) echo similar findings in existing literature. This study finds that the destination image of the tea estate can positively and significantly influence tourists’ flow experience, nostalgic feeling, and cultural identity. The empirical results of this study further confirm the applicability and validity of the studies on destination image by Kim (Kim and Hall, 2019), Shi (Shi et al., 2021), and Lee (Lee et al., 2021) in the field of tea estate tourism.

Second, regarding the relationship among tourists’ flow experience, nostalgic feeling, cultural identity, and behavioral intention, all hypotheses except H5 echo similar findings in existing literature (Brown, 2010; He and Wang, 2015; Bonaiuto et al., 2016; Fan et al., 2020). However, the empirical results do not suggest a significant correlation between tourists’ flow experience and their behavioral intention to tour the tea estate, a finding that contradicts the views of Zhou (Zhou, 2012) as well as some previous findings of other scholars (Hsu et al., 2012; Kazancoglu and Demir, 2021). This may be due to the trend of tea estate tourism being social-media-worthy following tourists’ flow experience. Tourism types such as social-media-worthy tourism are often superficial. In addition, the social-media-worthy effect is short-lived and unsustainable, often does not meet the continuous demand of tourists, and does not quickly generate intention to travel. It is very difficult for destinations to transition tourists into repeat visitors, and without repeat visitors, the flow of tourists to such destinations decreases sharply. Therefore, this study finds that tourists’ flow experience does not significantly enhance tourists’ behavioral intention.

Finally, we examine the mediating effects of flow experience, nostalgic feeling, and cultural identity and the moderating effect of cultural memory. In terms of the mediating effect, all mediating effect hypotheses hold except H10a (DI → FE → BI), largely because flow experience cannot significantly influence tourists’ behavioral intention, as described above. In terms of the moderating effect, all hypotheses are unsupported except H12b (DI → NF). The results from this study support that the integration of elements of cultural memory at tea estate tourism destinations can better fit tourists’ feelings towards products and can promote tourists’ nostalgia by stimulating their cultural memory. However, cultural memory focuses more on the generation of memories of the past without affecting the flow experience and cultural identity generated by tourists and thus has a more significant impact on tourists’ nostalgia.

### Theoretical contribution

First, this study enriches the research on destination image. Previous studies involving destination image have focused on heritage and cultural tourism destinations. With the increasing demand for cultivating new industries and new business forms of agriculture in rural areas, promoting the deep integration of rural industries, and continuously innovating new agritourism subjects, tea estate tourism as an innovative type of tourism has emerged and gradually become an important tourism destination for tourists (Mao et al., 2019). However, a review of previous studies reveals that few scholars have conducted research on agricultural estates that are both leisure tourism sites with agroecological environments and landscape structures and have cultural heritage (Lohmus and Liira, 2013). This study fills this gap by investigating the attitude and behavioral intention of tea estate tourists. Inspired by these important findings, future research can continue to explore the impact of tea estate image on tourists’ other psychological behaviors and intentions.

Second, this study enriches the research on tourists’ psychological perception. Psychological perception plays an important role in tourists’ travel experience. Some scholars have shown that tourists’ behavioral intention is further enhanced when they have a good flow experience, nostalgic feeling, and cultural identity (Zhou, 2012; Kessous et al., 2015; Zhang et al., 2020). However, there are very limited studies involving tourists’ psychological perception in either tea estate studies or agricultural estate studies. In particular, few studies have examined the dimensions of flow experience, nostalgic feeling, and cultural identity at the same time as well as the influence of tourists’ psychological perception on their subsequent behavior in tea estates.

Third, this study also contributes to the research on behavioral intention in agritourism and heritage tourism. Previous studies have shown that tourists’ post-trip behavior is significantly enhanced when they have a good tourism experience (Jin et al., 2013). Inspired by this finding, this study reconsiders the influence of this relationship on tourists’ behavior when they have a good flow experience, cultural identity, and nostalgic feeling.

Fourth, this study demonstrates the mechanism of the influence of tea estate image on tourists’ behavioral intention through an empirical analysis. The results of this study confirm the mediating effects of tourists’ behavioral intention on tea estate tourism and indicate that destination image can only influence tourists’ behavioral intention through nostalgia. Studies that use mediating variables such as flow experience, cultural identity, and nostalgic feeling have not been reported in the literature. This study explores the mediating role of internal states on behavioral intention and contributes to the literature by providing empirical support for this process. In addition, this study theorizes and empirically tests the moderating role of cultural memory. The results confirm that cultural memory has a significant positive moderating effect on the positive correlation between destination image and nostalgic feeling.

### Management implications

First, the results from this study indicate that when tourists acquire a good tourism destination image of a tea estate, their flow experience, cultural identity, and nostalgic feeling are significantly enhanced. Therefore, it is particularly important to build a good and unique tourism destination image of tea estates, which are core tourism destinations. Considering that destination image is mainly based on the facilities provided by tea estates and the personal experience of tourists, it is necessary for local governments and destination managers to develop tourism plans for tea estates based on scientific evidence to avoid homogeneity, simplification, and blind investment and to create a reasonable layout for a “unique brand for each estate” and a “unique brand for each area.”

Second, this study examines the influences of flow experience, cultural identity, and nostalgic feeling on tourists’ behavioral intention. The results indicate that cultural identity and nostalgic feeling have a significant impact on tourists’ post-trip behavioral intention. Furthermore, destination image can indirectly influence tourists’ behavioral intention through the partial mediating role of nostalgia, and influenced by destination image, nostalgic feeling is reinforced by the moderating effect of cultural memory. Considering that the dimensions of cultural identity and nostalgic feeling address the cultural connotation of tourists, local governments and destination managers should continue to consider improving the cultural products and services offered by tea estates. Therefore, tea estates should continuously improve the diversity of their experience programmes and, combined with regional culture, deeply explore the cultural connotation of Anxi Tieguanyin tea and transform resource advantages into brand advantages. For example, combining the multi-cultural characteristics of Quanzhou, such as the world cultural heritage of Quanzhou: Emporium of the World in Song-Yuan China and the construction of the GIAHS: Anxi Tieguanyin Tea Culture System, the regional culture, Maritime Silk Road culture, tea culture and tea industry natural landscape (terroir and ecology), cultural landscape (tea estate and garden), and social culture (tea tasting, tea processing, tea making, tea recipes, tea clothing, folk customs, art, and religion) are organically integrated and should be explored to create tourism attractions and differentiation. Additionally, it is necessary to make good use of intelligent tourism information platforms for cultural communication to enhance brand image.

Third, the results from this study indicate that flow experience has no influence on tourists’ behavioral intention and that flow experience can indirectly influence behavioral intention only through the mediating effect of cultural identity. Therefore, in the Internet era, it is necessary to promote the concept, innovate the processes, optimize the structure, and improve the mechanisms of tourism governance. While making good use of “emotional resonance” and “relational identity,” tourist volume should be considered to avoid over-marketing and over-commercialization so that the value of tourism destinations can gain build through flow in society. Themed activities, entertainment facilities, and transportation should be reorganized and improved to ensure tourist safety so that tourists have a sufficient sense of identity and satisfaction, which may effectively improve tourists’ intention to travel.

### Limitations and future research

This study has two major limitations. First, the sample includes mainly Chinese tourists. Therefore, a larger sample size from different countries and regions should be used in future studies to explore their attitudes towards tea estate tourism destinations. Second, different countries have different cultural and landscape tourism contexts; this study may only be applicable to domestic research on relevant agricultural industries in China but not to research on foreign-related agricultural industries. Therefore, based on the inspiration of this study, future research can explore more consumer perception behavior of agricultural estate tourism.

## Data availability statement

The datasets presented in this study can be found in online repositories. The names of the repository/repositories and accession number(s) can be found in the article/Supplementary material.

## Author contributions

SZ, JL, YM, YC, and QH participated in the design, documentation, development, and writing of the manuscript, reviewed the article, were responsible for its contents, and provided responsible for the final version. All authors contributed to the article and approved the submitted version.

## Funding

Projects Supported By National Social Science Foundation “Temporal and spatial differentiation law and management response of ecological tourism industry in China” (21BGL148); Project supported by the Ministry of Agriculture and Rural Affairs of the People’s Republic of China: Collaborative Innovation Center Project of Modern Agricultural Industrial Park in Anxi County, Fujian Province (KMD18003A); 2021 Young and Middle-aged Teachers’ Education and Scientific Research Project (Social Science) General Project “Research on the Innovative Path of Traditional Festivals to Enhance cultural Identity” (Project Number JAS21438); Funded by the Innovation Strategy Research Program of Fujian Province, research on collective memory construction and living protection of Fujian tea cultural heritage (2021R0039).

## Conflict of interest

The research was conducted in the absence of any commercial or financial relationships that could be construed as a potential conflict of interest.

## Publisher’s note

All claims expressed in this article are solely those of the authors and do not necessarily represent those of their affiliated organizations, or those of the publisher, the editors and the reviewers. Any product that may be evaluated in this article, or claim that may be made by its manufacturer, is not guaranteed or endorsed by the publisher.

## Appendix

Background of respondents (*N* = 277).

**Table:**

	Item	Frequency	Percent (%)
Gender	Males	143	51.6
	Females	134	48.4
Age	18–30	202	72.9
	31–40	51	18.4
	41–50	18	6.5
	51–60	3	1.1
	Over 60	3	1.1
Education	High School or below	25	8.1
	Junior College	22	7.9
	Undergraduate	203	73.3
	Postgraduate	27	9.7
Income Individual monthly	CNY 4000 or below	107	38.6
	CNY 4001–6,000	59	21.3
	CNY 6001–8,000	46	16.6
	CNY 8001–10,000	25	9.0
	Over CNY 15000	40	14.4
Job	Student	159	57.4
	Worker/Staff	53	19.1
	Public Institution Employees	6	2.2
	Retirees	2	2.5
	Other Staff	57	20.6
